# Morquio A syndrome associated with bilateral hydroureteronephrosis and neurogenic bladder: a rare case report and review of the literature

**DOI:** 10.1186/s12887-026-07330-3

**Published:** 2026-07-25

**Authors:** Bablu Kumar Gaur, Shreya Gupta, Ishfaq Ahmad Pir, Shreyashi Jain

**Affiliations:** Department of Pediatrics, Teerthanker Mahaveer Medical College, and Research Center, TMU, Moradabad, U.P 244001 India

**Keywords:** Morquio A syndrome, Hydroureteronephrosis Neurogenic bladder, MRI spine

## Abstract

**Background:**

Morquio syndrome is a lysosomal storage disorder that damages several organs due to mucopolysaccharide accumulation in cells and tissues. Here, we highlight a case of Morquiosyndrome associated with hydroureteronephrosis and neurogenic bladder.

**Case presentation:**

An 8-year-old girl presented with a 5-year history of skeletal deformities, followed by urinary dribbling, nocturnal enuresis, and intermittent fever for the past 6 months. Physical examination revealed classical features of mucopolysaccharidosis, a suprapubic bulge, and lower limb weakness. Urine microscopy demonstrated pyuria. Abdominal ultrasonography showed bilateral gross hydroureteronephrosis with an overdistended bladder. Magnetic resonance urography confirmed bilateral gross hydroureteronephrosis with dilated, tortuous ureters. MRI of the whole spine revealed severe spinal canal stenosis at C2–3 and D10–11, along with platyspondyly. Enzyme assay demonstrated a markedly reduced level of N-acetylgalactosamine-6-sulfatase. Due to financial constraints, the parents declined spinal surgery. The patient was managed with clean intermittent self-catheterization (CISC) for neurogenic bladder.

**Conclusion:**

Hydroureteronephrosis and neurogenic bladder are rarely associated with Morquio syndrome. Clinicians should be aware of this rare association with mucopolysaccharidosis. Given that early identification can greatly lower morbidity and death in these individuals. Spinal imaging is necessary in all the case of Morquio syndrome A.

## Background

Morquio syndrome is a mucopolysaccharidosis (MPS) caused by a deficiency of the enzyme *N*-acetylgalactosamine-6-sulfatase [1]. This enzymatic defect results in impaired lysosomal degradation of keratan sulfate and chondroitin-6-sulfate, leading to their accumulation and subsequent dysfunction of cells, tissues, and multiple organs. Patients with MPS typically exhibit abnormalities of connective tissue, cartilage, ligaments, and bone [2]. They are often identified in early childhood due to prominent skeletal deformities. Characteristic features include disproportionately short stature, atlantoaxial instability, odontoid hypoplasia, platyspondyly, kyphoscoliosis, pectus carinatum, coxa valga, and genu valgum [3]. Severe spinal canal stenosis and atlantoaxial subluxation may predispose patients to cervical myelopathy, resulting in complications such as neurogenic bladder and hydroureteronephrosis [4]. While most research on Morquio syndrome has emphasized progressive skeletal dysplasia, only a few case reports have described its effects on the spinal cord and urinary function, including neurogenic bladder and incontinence [5, 6]. However, to date, no reports have documented mucopolysaccharidosis associated with bilateral gross hydroureteronephrosis. We present an unusual case of an 8-year-old child with classical features of Morquio syndrome, complicated by bilateral gross hydroureteronephrosis and neurogenic bladder.

## Case presentation

**Fig. 1:**
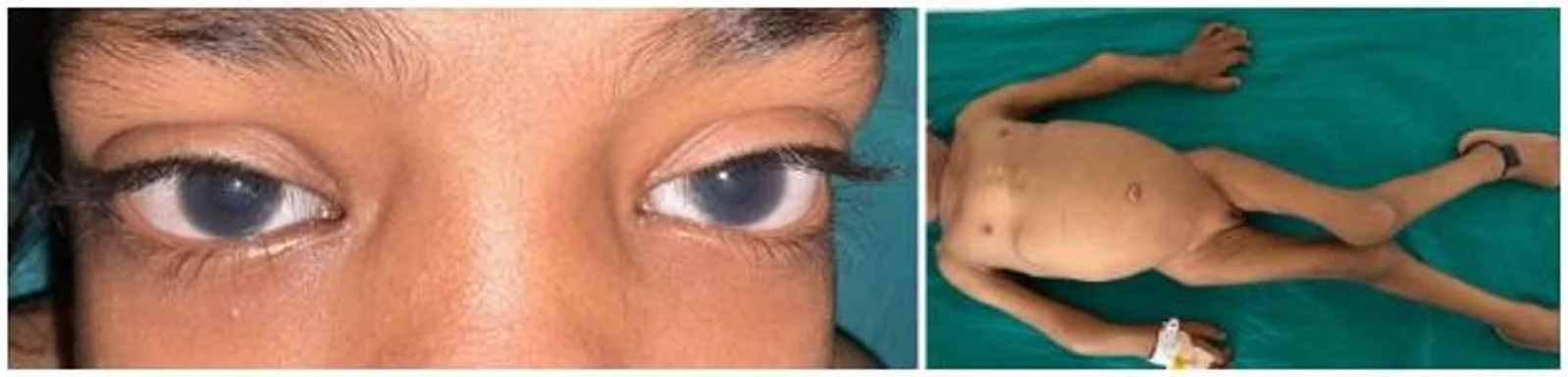
Female child diagnosed with Morquio syndrome having, coarse facies, corneal opacity, short neck, low hairline, pigeon chest, severe knock knee, and kyphoscoliosis

**Fig. 2:**
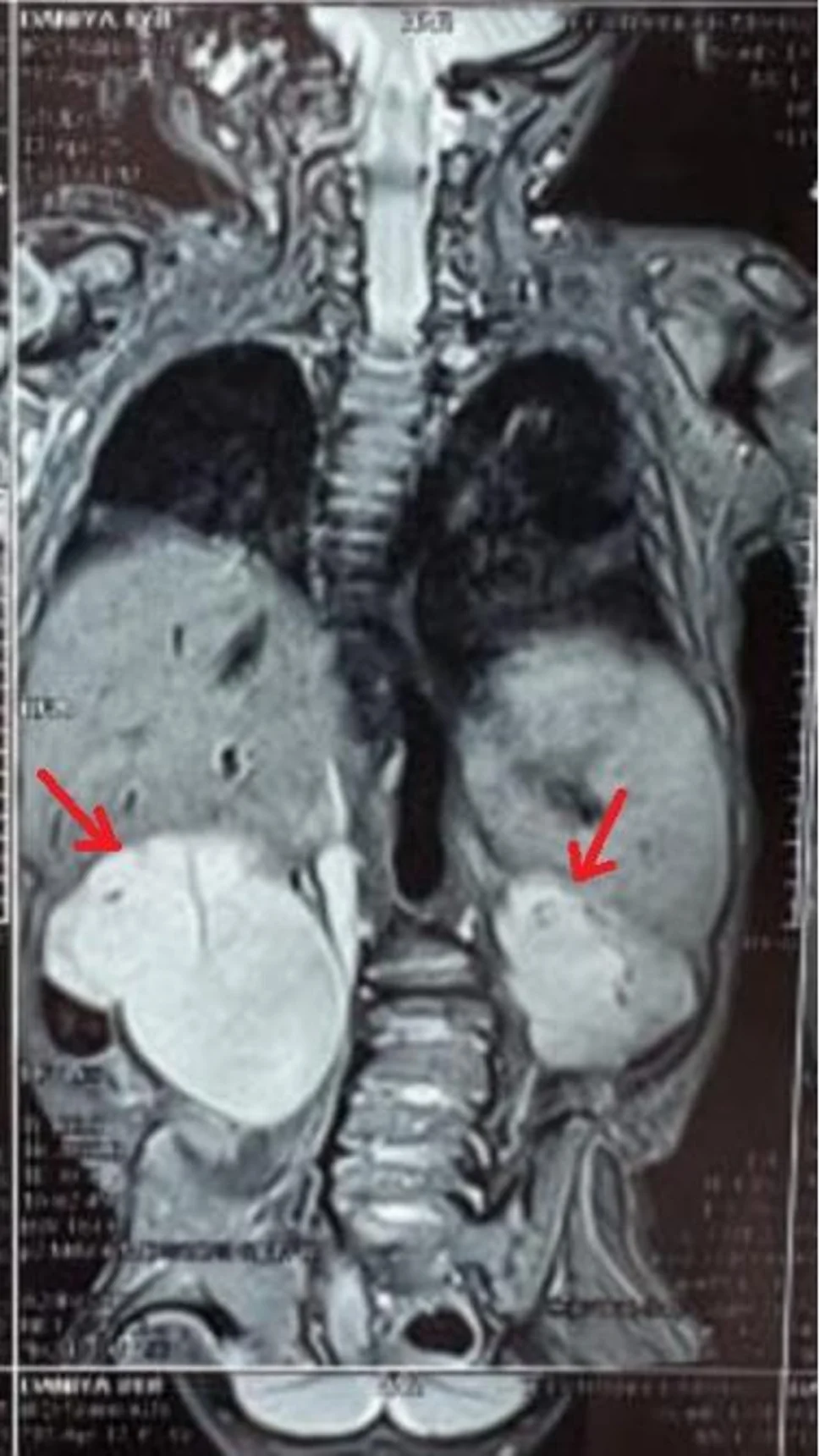
MR urography showing bilateral gross hydronephrosis with grossly dilated and tortuous bilateral ureters

**Fig. 3:**
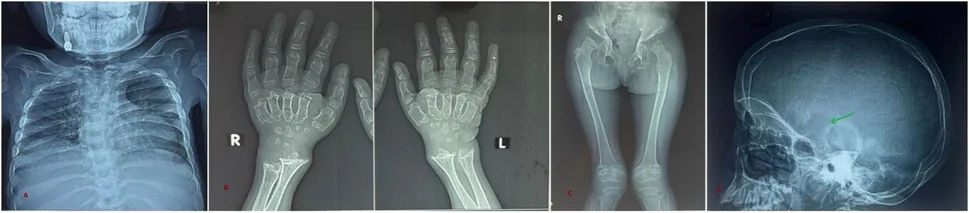
**A** The radiographs of the chest showing kyphoscoliosis, oar-shaped ribs, and anterior vertebral beaking. **B** X-ray of lower limb showing osteopenia along with poorly formed and fragmented femur and epiphyseal ends of tibia and fibula. **C** X-ray of the wrist showing bullet-shaped metacarpals. **D** X-ray Skull lateral view showing J-shaped sella turcica (red arrow)

**Fig. 4:**
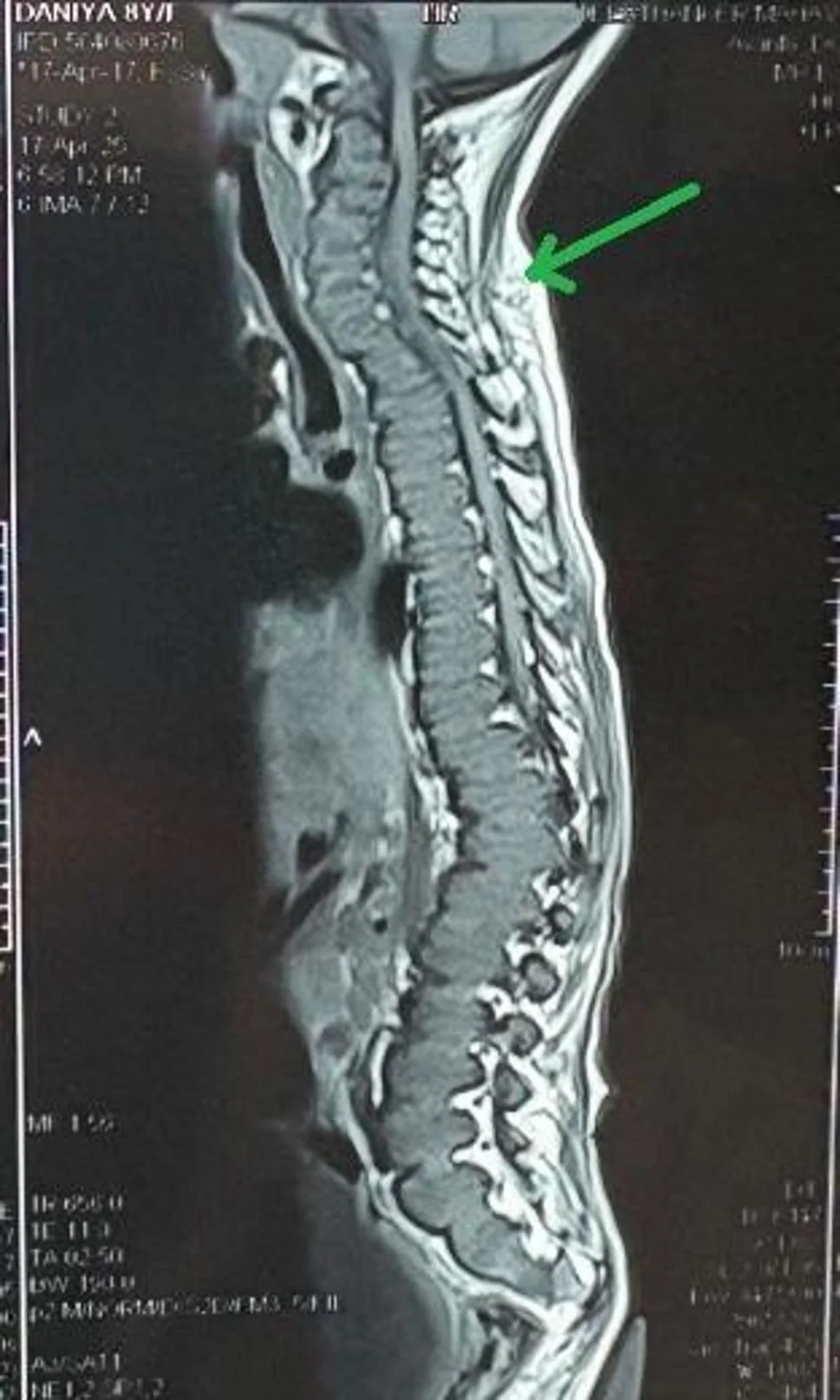
MRI whole spine sagittal view showing platyspondyly with severe spinal canal stenosis at C2-3 and D10-11 level with myelopathic changes (green arrow)

An 8-year-old female child, born to a non-consanguineous couple, presented with a 6-year history of progressive skeletal deformities. At 6 years of age, she had experienced progressive lower limb weakness (paraplegia), which eventually led to wheelchair dependence. At the age of 7 years, she developed urinary dribbling, nocturnal enuresis, and recurrent fever. The parents also reported gradually worsening wrist deformity (widening with hypermobility) and chest deformity (pectus carinatum) over the preceding 5–6 years. There was no family history of hereditary disorders. Antenatal and birth history were unremarkable. On admission, her vital signs were: temperature 39.2 °C, heart rate 132/min, blood pressure 104/72 mmHg, respiratory rate 22/min, and oxygen saturation 97% on room air. General examination revealed disproportionate short stature (upper segment shorter than lower segment), coarse facies, short neck with low hairline, dorsolumbar kyphoscoliosis, pectus carinatum, brittle teeth, genu valgum, hypermobility of wrist joints, and ankle contractures (Fig. 1). Anthropometric measurements, plotted on WHO growth charts, showed a height of 87 cm (< 3rd centile), weight of 11 kg (< 3rd centile), and body mass index of 14.1 (10th–50th centile). Arm span was 88 cm, and the upper-to-lower segment ratio was 0.75. Neurological examination revealed power of 0/5 in the lower limbs and 5/5 in the upper limbs, brisk deep tendon reflexes with ankle clonus, and intact cranial nerves. She was conscious, well oriented (GCS 15/15), and had normal fundus findings. Developmental assessment showed normal scholastic performance and IQ of 89. Abdominal examination revealed mild distension with suprapubic bulge and flank fullness. Laboratory investigations showed hemoglobin 10.2 g/dL (reference 11–14 g/dL), total leukocyte count 19.6 × 10⁹/L (81% neutrophils, 14% lymphocytes), platelet count 2.3 × 10⁹/L (reference 1.5–4.0 × 10⁹/L), C-reactive protein 47 mg/L (reference 0–6 mg/L), and ESR 18 mm/hr (reference 0–20 mm/hr). Urine microscopy revealed 25 pus cells/HPF (reference 0–5/HPF). Renal function tests were within normal limits. Peripheral smear showed microcytic anemia with mild anisopoikilocytosis. A provisional diagnosis of mucopolysaccharidosis with urinary tract infection (UTI) was made. She was started on intravenous cefotaxime (500 mg every 12 h), amikacin (75 mg every 12 h), urinary catheterization, and supportive therapy. Blood and urine cultures were sterile. Abdominal ultrasonography revealed bilateral gross hydroureteronephrosis with an overdistended bladder. Magnetic resonance urography confirmed bilateral gross hydronephrosis (anteroposterior diameter of renal pelvis: 32 mm on right, 16 mm on left) with grossly dilated, tortuous ureters (11 mm bilaterally) (Fig. 2). The bladder was overdistended with irregular walls and trabeculations. Micturating cystourethrogram (MCU) could not be performed due to active UTI. Skeletal survey revealed multiple dysplastic changes: chest X-ray showed dorsal scoliosis with oar-shaped ribs (Fig. 3A); lower limb X-ray revealed osteopenia and poorly formed, fragmented femoral epiphyses with dysplastic tibial and fibular epiphyses (Fig. 3B); wrist X-ray showed bullet-shaped metacarpals, medial slanting of metaphyseal ends of radius and ulna, and poorly formed carpal epiphyses (Fig. 3C); lateral skull X-ray revealed a J-shaped sella turcica (Fig. 3D). MRI of the whole spine demonstrated platyspondyly and severe spinal canal stenosis at C2–3 and D10–11, with myelopathic changes (Fig. 4). Enzyme assay confirmed markedly reduced *N*-acetylgalactosamine-6-sulfatase activity (1.2 nmol/L; reference 24.44–216.69 nmol/[17 h·mg]). Genetic testing could not be performed due to financial limitations. Whole- exome sequencing identified a homozygous GLANS gene mutation , c.107T>G (p.Leu 36Arg). Adiagnosis of Morquio A syndrome associated with bilateral gross hydroureteronephrosis and neurogenic bladder was established. The parents declined spinal surgery due to financial constraints. The child was managed with clean intermittent self-catheterization (CISC). The disease course, prognosis, and long-term care plan were explained to the family.

## Discussion

Morquio syndrome is an autosomal recessive mucopolysaccharidosis characterized by deficiency of specific lysosomal enzymes required for degradation of acidic mucopolysaccharides [1]. Two variants are described: type A, caused by deficiency of *N*-acetylgalactosamine-6-sulfatase (GALNS) due to mutations in the gene located at 16q24.3, and type B, resulting from absence of β-galactosidase [2]. In both forms, keratan sulfate and chondroitin-6-sulfate accumulate, leading to multi-system involvement. The hallmark clinical features include short stature, short trunk, short neck, corneal opacity, pectus carinatum, wrist broadening, kyphoscoliosis, ligamentous laxity, and genu valgum [3]. In our case, most of these classical manifestations were present. A key distinction from other mucopolysaccharidoses (MPS) is the preservation of normal intelligence, attributed to intact metabolism of heparan and dermatan sulfates [7]. Although the disorder predominantly affects the skeletal system, involvement of the skin, kidneys, urinary bladder, and central nervous system is rare [3]. Neurogenic bladder dysfunction in Morquio syndrome results from compression of the spinal cord due to severe spinal canal stenosis or atlantoaxial instability. While the precise mechanism remains unclear, one hypothesis suggests disruption of the autonomic innervation of the bladder: sympathetic input (T10–L2) normally contracts the bladder neck and inhibits detrusor muscle activity, whereas parasympathetic input (S2–S4) stimulates detrusor contraction and relaxes the urethral sphincter [8]. Budak et al. proposed that urinary glycosaminoglycan excretion may serve as a marker of bladder wall injury and degeneration in MPS patients with neurogenic bladder [9]. Only a few reports have described urinary bladder dysfunction, such as incontinence and neurogenic bladder, in Morquio syndrome [5, 6]. Ge et al. reported two boys (aged 10 and 18 years) with classical features of Morquio syndrome, urinary incontinence, lower limb paralysis, and normal IQ; however, neither patient exhibited vesicoureteric reflux or gross hydroureteronephrosis [5]. Koyama et al. described neurogenic bladder secondary to cervical myelopathy in a patient with Hunter syndrome [6]. Remondino et al. reported spinal involvement in 52 MPS patients, of whom 21 developed neurologic compromise, most commonly quadriparesis [10]. The present case is unusual in that the child developed bilateral gross hydroureteronephrosis secondary to neurogenic bladder, complicated by urinary tract infection. To the best of our knowledge, this association has not been reported previously. Enzyme replacement therapy (ERT) remains the standard treatment for Morquio syndrome. Although ERT does not reverse skeletal deformities, it slows disease progression and improves systemic outcomes, particularly when initiated in the pre-symptomatic stage. Limitations include its high cost and limited availability in low-resource settings.

## Conclusions

Children with Morquio syndrome may develop gross hydroureteronephrosis secondary to neurogenic bladder. For early detection of these complications, all patients should undergo detailed history-taking, thorough clinical examination, magnetic resonance urography, and whole-spine MRI.

## Data Availability

The data that support the findings of this study are available from the corresponding author upon reasonable request.

## Abbreviations

MPS: Mucopolysaccharidosis
GALNS: N acetylgalactosamine-6-sulfate sulfatase
MRI: Magnetic resonance spectroscopy
WHO: World health organization
IQ: Intelligence quotient
CKD: Chronic kidney disease
VUR: Vesico-ureteric reflex GCS, Glasgow coma scale
HDN: hydroureteronephrosis
MR: Magnetic resonance
